# Clinical Decision-Making After Receptor Conversion Following Neoadjuvant Therapy in Breast Cancer: A Three-Strategy Framework

**DOI:** 10.3390/jcm15187141

**Published:** 2026-09-15

**Authors:** Katarzyna Pogoda, Magdalena Czopowicz, Wojciech Olszewski, Monika Durzynska, Piotr Jan Wysocki

**Affiliations:** 1 Department of Breast Cancer and Reconstructive Surgery, Maria Sklodowska-Curie National Research Institute of Oncology, Roentgena 5, 02-781 Warsaw, Poland; 2 National Cancer Strategy Department, Maria Sklodowska-Curie National Research Institute of Oncology, Roentgena 5, 02-781 Warsaw, Poland; 3 Department of Pathology, Maria Sklodowska-Curie National Research Institute of Oncology, Roentgena 5, 02-781 Warsaw, Poland; 4 Department of Oncology, Jagiellonian University Medical College, Kopernika 50, 31-501 Krakow, Poland

**Keywords:** breast cancer, neoadjuvant therapy, receptor conversion, residual disease, biomarkers, clinical decision-making

## Abstract

**Background:** Receptor conversion following neoadjuvant therapy may alter eligibility for adjuvant systemic therapy in breast cancer. However, current guidelines provide limited recommendations for biomarker-guided treatment adaptation after receptor change. We aimed to characterize clinical decision-making after receptor conversion and identify a framework describing treatment adaptation across clinically relevant receptor conversion scenarios. **Methods:** A nationwide cross-sectional survey was conducted between August and September 2025 among Polish medical oncologists involved in breast cancer care. The questionnaire assessed receptor reassessment practices and treatment recommendations across five receptor conversion scenarios. Treatment decisions were classified into three predefined strategies—baseline-driven, residual disease-driven, and combined—according to whether they relied on pretreatment biomarkers, residual disease biomarkers, or both. **Results:** A total of 104 medical oncologists completed the survey. Routine receptor reassessment in residual disease was reported by 52.9% of respondents, while 73.1% incorporated residual disease biomarkers into treatment planning either routinely or selectively. Overall, baseline-driven strategies accounted for only 26.3% of treatment decisions, compared with 40.2% for combined strategies and 33.5% for residual disease-driven strategies. Combined strategies predominated following hormone receptor conversion (52.9% for luminal HER2-negative to TNBC and 55.8% for TNBC to luminal HER2-negative). Baseline-driven approaches were most frequently selected following HER2 loss (42.3%), whereas HER2 gain favored residual disease-driven strategies in TNBC-to-HER2-positive conversion (46.2%) and combined strategies in luminal HER2-negative-to-HER2-positive conversion (48.1%). **Conclusions:** A three-strategy framework of clinical decision-making emerged after receptor conversion, with most treatment decisions incorporating residual disease biology despite limited guideline direction. This framework highlights an important evidence gap and provides a practical foundation for future studies evaluating treatment adaptation after receptor conversion.

## 1. Introduction

Neoadjuvant therapy has become a standard component of breast cancer management, particularly in patients with locally advanced or biologically aggressive disease [1]. Although pathological complete response is associated with favorable outcomes, many patients have residual disease requiring post-neoadjuvant treatment decisions with important prognostic implications [2].

Emerging evidence indicates that the biological characteristics of residual disease may differ from those of the primary tumor, a phenomenon referred to as receptor conversion [3]. Changes in the expression of estrogen receptor (ER), progesterone receptor (PR), and human epidermal growth factor receptor 2 (HER2) have been reported in up to 40% of cases, depending on the biomarker and clinical context [4,5]. These changes may alter eligibility for adjuvant systemic therapy, including endocrine therapy, anti-HER2 treatment, and chemotherapy intensification.

Despite increasing recognition of receptor conversion, its clinical implications remain uncertain. Current international guidelines do not address routine biomarker reassessment in residual disease or provide clear recommendations for treatment adaptation [1,6,7,8,9]. As a result, current management practices may vary, including both receptor reassessment and subsequent therapeutic decisions.

Furthermore, the post-neoadjuvant treatment landscape has become increasingly complex, with the availability of therapeutic options, including CDK4/6 inhibitors, olaparib (PARP inhibitor), and trastuzumab emtansine (T-DM1) [1,7]. As treatment options expand, clinicians must decide whether baseline biology, residual disease biology, or both should guide treatment despite the absence of standardized guidance.

Importantly, while previous studies have described variability in biomarker reassessment [10,11], little is known about how oncologists translate receptor change into real-world treatment decisions. In the absence of prospective evidence supporting most receptor conversion scenarios, clinicians must rely largely on clinical judgment. Consequently, it remains unclear whether treatment decisions are based on baseline tumor characteristics, residual disease biology, or both.

To better understand current patterns of clinical decision-making in this setting, we conducted a nationwide survey of medical oncologists involved in breast cancer care in Poland. Specifically, we evaluated receptor reassessment practices and characterized treatment strategies across clinically relevant receptor conversion scenarios.

## 2. Methods

### 2.1. Study Design and Participants

This nationwide cross-sectional survey was conducted between August and September 2025 under the auspices of the Polish Society of Clinical Oncology (PTOK). The questionnaire was distributed during the annual PTOK congress and subsequently through professional electronic mailing lists. Participation was voluntary and anonymous. Only completed questionnaires from medical oncologists involved in breast cancer treatment were included in the analysis.

### 2.2. Survey Development

The questionnaire was developed by a multidisciplinary team of breast cancer specialists, including medical oncologists and pathologists, to reflect clinically relevant challenges in managing residual disease following neoadjuvant therapy. The five receptor-conversion scenarios were selected to represent clinically plausible phenotype changes with the potential to influence post-neoadjuvant treatment. Individual treatment components were based on contemporary guideline-supported therapies for the relevant baseline or residual disease phenotypes. Because direct evidence is unavailable for most receptor-conversion scenarios, the proposed treatment combinations were refined through multidisciplinary expert review and were intended to elicit clinical reasoning rather than assess guideline concordance. Draft questions were reviewed by the study team to ensure clinical relevance, clarity, and consistency with contemporary post-neoadjuvant treatment decision-making.

The final instrument consisted of 15 questions organized into four domains: (1) respondent and institutional characteristics, (2) institutional practices in receptor reassessment in residual disease, (3) treatment decision-making in predefined clinical scenarios, and (4) additional factors influencing postoperative treatment decisions. The full questionnaire is provided in the Supplementary Materials.

### 2.3. Survey Content

Participants provided information on clinical experience, institutional volume, and practice setting, including affiliation with a certified Breast Cancer Unit. Current practice patterns were assessed by asking about routine evaluation of receptor status (ER, PR, and HER2) in residual disease and the extent to which these findings influence treatment decisions.

Five case-based scenarios representing clinically relevant receptor conversion patterns were developed to evaluate post-neoadjuvant treatment decision-making across transitions between triple-negative, luminal HER2-negative, and HER2-positive disease. Each scenario incorporated key clinicopathologic features reflecting routine clinical practice, and respondents were asked to select the treatment approach most consistent with their usual practice.

Additional questions assessed the role of tumor grade and Ki-67 in residual disease in treatment decision-making, particularly their influence on the use of capecitabine in hormone receptor-positive residual disease.

### 2.4. Definition of Treatment Strategies

To characterize clinical decision-making, responses to case-based scenarios were categorized into three predefined strategies: (1) baseline-driven approaches, reflecting treatment decisions based on the initial tumor phenotype; (2) combined approaches, integrating baseline and residual disease characteristics; and (3) residual disease-driven approaches, reflecting treatment decisions based primarily on the receptor phenotype in residual disease. The classification was developed based on contemporary therapeutic principles and international guideline recommendations (American Society of Clinical Oncology [ASCO], European Society for Medical Oncology [ESMO], and National Comprehensive Cancer Network [NCCN]) while recognizing that prospective evidence is lacking for most receptor conversion scenarios [1,6,7,12,13]. In Poland, NCCN Guidelines are incorporated into national harmonized recommendations and are routinely used in clinical practice. The framework was designed to compare decision-making patterns rather than define any strategy as evidence-based or guideline-concordant. Individual treatment options were classified into three predefined decision-making strategies (Table 1).

### 2.5. Statistical Analysis

Descriptive statistics summarized respondent characteristics and survey responses. Categorical variables are presented as frequencies and proportions (%). Given the exploratory nature of the study, no confirmatory hypotheses were specified a priori, and analyses primarily focused on identifying patterns of variability and consensus in clinical decision-making.

Proportions of respondents selecting particular strategies within and across clinical scenarios were compared using significance tests for paired (dependent) groups—McNemar’s test with the Yates correction for continuity for comparing two proportions and Cochran’s Q test for comparing three or more proportions. A significance level (α) was set at 0.05, and a Bonferroni correction was applied for multiple comparisons. Because responses across clinical scenarios were provided by the same participants, paired tests were used to account for within-respondent correlation. These exploratory analyses assessed whether selection proportions differed across strategies or scenarios within the surveyed sample. Statistical analysis was conducted in TIBCO Statistica 13.3 (TIBCO Software Inc., Palo Alto, CA, USA).

## 3. Results

### 3.1. Respondent Characteristics

A total of 104 medical oncologists involved in breast cancer treatment completed the survey. Respondents represented a broad range of experience levels and practice settings. Most respondents were experienced clinicians, with 55 (52.9%) reporting more than 10 years of practice, 24 (23.1%) reporting 5–10 years, and 25 (24.0%) reporting less than 5 years of experience. Respondents also represented centers with varying breast cancer volumes, with 27 (26.0%) reporting 500 to 1000 cases per year, 21 (20.2%) reporting 250 to 500 cases, and smaller proportions representing both lower- and higher-volume centers. Overall, 62.5% of respondents practiced within a dedicated Breast Cancer Unit (Table 2).

### 3.2. Receptor Reassessment and Its Influence on Treatment Decisions

Routine reassessment of ER, PR, and HER2 in residual disease following neoadjuvant therapy was reported by 52.9% of respondents (Table 3). Receptor reassessment was more common among respondents working in dedicated breast cancer units (40/65 [61.5%] vs. 15/39 [38.5%]; *p* = 0.023), in centers managing >1000 newly diagnosed breast cancer cases annually (10/12 [83.3%] vs. 45/92 [48.9%]; *p* = 0.025), and clinicians with more than 10 years of practice (36/55 [65.5%] vs. 19/49 [38.8%]; *p* = 0.007).

Incorporation of residual tumor biology into treatment decisions was reported consistently by 47 respondents (45.2%) and selectively by 29 respondents (27.9%), indicating that residual disease findings influenced treatment planning even though routine reassessment was not universal.

**Figure 1:**
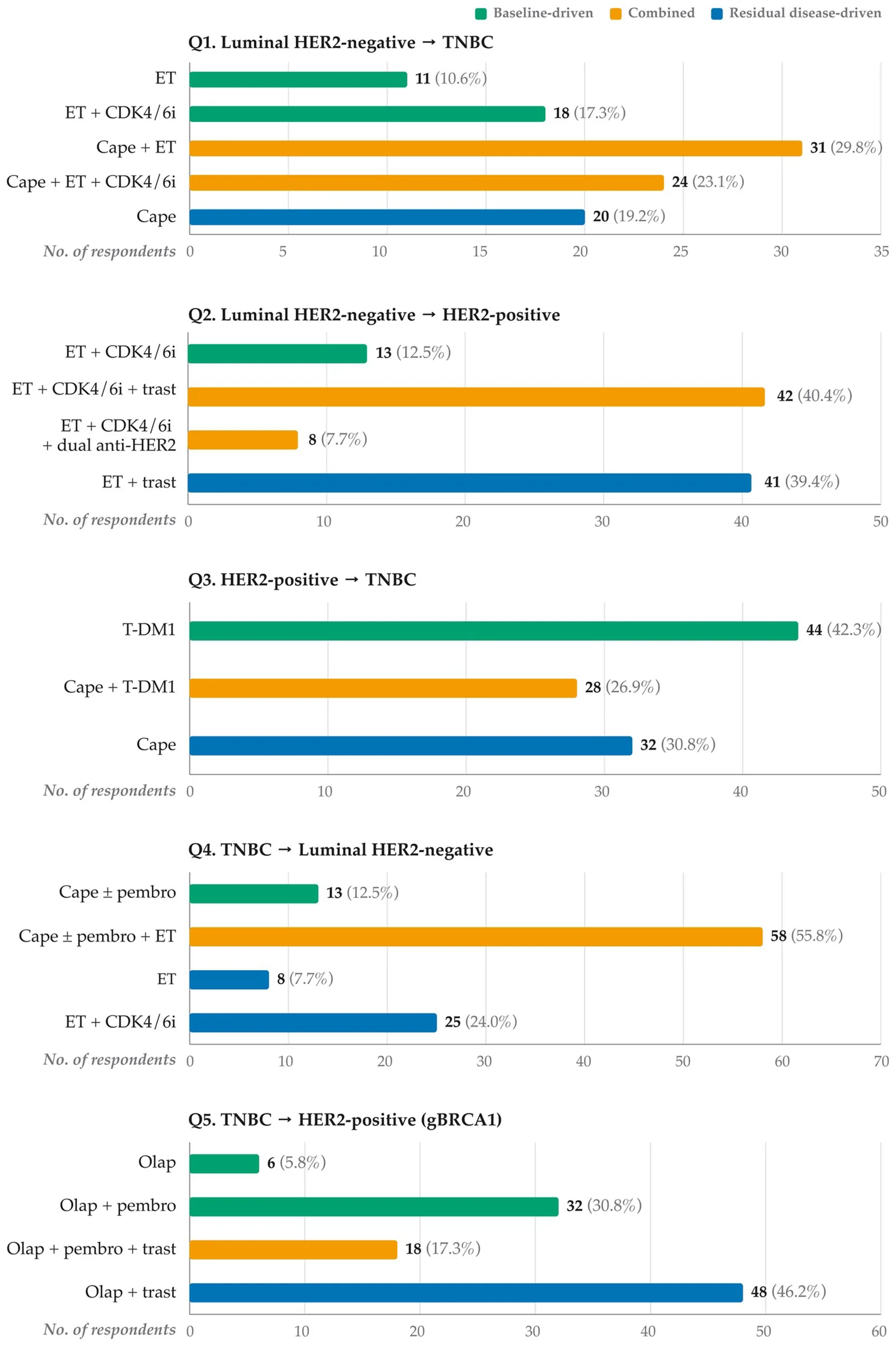
Treatment preferences across five case-based receptor-conversion scenarios: Q1, luminal HER2-negative to TNBC; Q2, luminal HER2-negative to HER2-positive; Q3, HER2-positive to TNBC; Q4, TNBC to luminal HER2-negative; Q5, TNBC to HER2-positive in a germline BRCA mutation carrier. Bars show the number and percentage of respondents selecting each treatment option; colors denote the predefined treatment strategy: baseline-driven, combined, or residual disease-driven. Abbreviations: Cape, capecitabine; CDK4/6, cyclin-dependent kinase 4 and 6; ET, endocrine therapy; HER2, human epidermal growth factor receptor 2; *gBRCA*—germline *BRCA* mutation; Olap, olaparib; pembro, pembrolizumab; T-DM1, trastuzumab emtansine; TNBC, triple-negative breast cancer; trast, trastuzumab; dual anti-HER2 therapy, trastuzumab + pertuzumab.

Clinical scenarios are illustrated in Figure 1, and treatment options were categorized into three predefined strategies—baseline-driven, combined, and residual disease-driven—as described in Methods (Table 1). These categories were used to characterize treatment decision-making across receptor conversion scenarios.

### 3.3. Q1. Luminal HER2-Negative to TNBC Conversion

In the setting of luminal HER2-negative tumors converting to a triple-negative breast cancer (TNBC) phenotype, combined strategies were selected by the majority of respondents (52.9%), followed by baseline-driven approaches (27.9%) and residual disease-driven strategies (19.2%) (Figure S1). Capecitabine combined with endocrine therapy was the most commonly selected option. This combination reflected a reported approach that preserved endocrine therapy while adding treatment directed toward the residual TNBC phenotype.

### 3.4. Q2. Luminal HER2-Negative to HER2-Positive Conversion

In the scenario of luminal tumors converting from HER2-negative to HER2-positive disease, baseline-driven approaches were selected least often (12.5%). Respondents most often selected either combined strategies (48.1%) or residual disease-driven approaches based on the HER2-positive phenotype (39.4%) (Figure S1). Two regimens were selected with similar frequency: endocrine therapy combined with a CDK4/6 inhibitor and trastuzumab (40.4%) and endocrine therapy with trastuzumab alone (39.4%). HER2 gain appeared to influence treatment selection, with most respondents selecting strategies that incorporated HER2-directed therapy.

### 3.5. Q3. HER2-Positive to TNBC Conversion

In contrast, when HER2-positive disease converted to a triple-negative phenotype, baseline-driven strategies were most common (42.3%), followed by residual disease-driven approaches (30.8%) and combined strategies (26.9%) (Figure S1). T-DM1 was the most common treatment in this scenario. In contrast to HER2 gain, HER2 loss did not uniformly lead respondents to abandon HER2-directed post-neoadjuvant therapy.

### 3.6. Q4. TNBC to Luminal HER2-Negative Conversion

In TNBC converting to a luminal HER2-negative phenotype, a majority of respondents selected combined strategies (55.8%). Residual disease-driven approaches were selected by 31.7% of respondents, while baseline-driven strategies were selected by 12.5% (Figure S1). Capecitabine combined with pembrolizumab and endocrine therapy was most commonly selected. The most common responses combined endocrine therapy with treatment elements associated with the baseline TNBC phenotype.

### 3.7. Q5. TNBC to HER2-Positive Conversion with BRCA Mutation

In the setting of TNBC converting to HER2-positive disease in the context of a germline *BRCA* mutation, residual disease-driven approaches were selected by 46.2% of respondents, followed by baseline-driven strategies (36.5%) and combined strategies (17.3%) (Figure S1). Olaparib in combination with trastuzumab was the most frequently selected option. The distribution of responses reflected the complexity of weighing HER2 gain, baseline TNBC biology, and germline *BRCA* status in a single post-neoadjuvant scenario.

### 3.8. Use of Additional Pathological Factors

Beyond receptor status, a majority of clinicians reported incorporating additional pathological features into treatment decisions. Tumor grade in residual disease was considered by 67.3% of respondents, and Ki-67 by 68.3%. Consistent with this, the use of capecitabine in the post-neoadjuvant setting varied: 14% of respondents reported routine use in luminal HER2-negative breast cancer, 26% reported not using capecitabine in this context, and approximately 60% indicated selective use based on pathological features such as tumor grade and Ki-67 in residual cancer tissue. Together, these findings indicate that treatment adaptation was influenced by broader residual disease features and not receptor status alone.

### 3.9. Summary of Decision-Making Patterns

Across all receptor conversion scenarios, respondents demonstrated three distinct patterns of clinical decision-making based on the predefined treatment strategy classification. Overall, combined strategies were selected most frequently (40.2% of 520 scenario-specific responses), followed by residual disease-driven (33.5%) and baseline-driven approaches (26.3%). The distribution of treatment strategies differed significantly across clinical scenarios in exploratory within-respondent comparisons (*p* < 0.001).

Baseline-driven strategies were most frequently observed in HER2-positive to triple-negative conversion (42.3%) and were least commonly selected in luminal HER2-negative to HER2-positive and TNBC to luminal HER2-negative conversion (both 12.5%; Figure S2). This pattern suggests that respondents were more likely to preserve baseline-directed treatment after HER2 loss, whereas acquisition of a potentially actionable receptor was associated with greater use of residual disease-driven or combined strategies.

Combined strategies reached their highest use in TNBC to luminal HER2-negative conversion (55.8%) and were also frequently selected in luminal HER2-negative to TNBC change (52.9%) or luminal HER2-negative to HER2-positive conversion (48.1%). Their use was lower in HER2-positive to TNBC conversion (26.9%) and TNBC to HER2-positive change with a germline *BRCA* mutation (17.3%; Figure S3). Across scenarios, combined strategies appeared to function as a pragmatic approach to uncertainty, allowing respondents to retain elements of baseline-directed treatment while also responding to the phenotype of residual disease.

**Figure 2:**
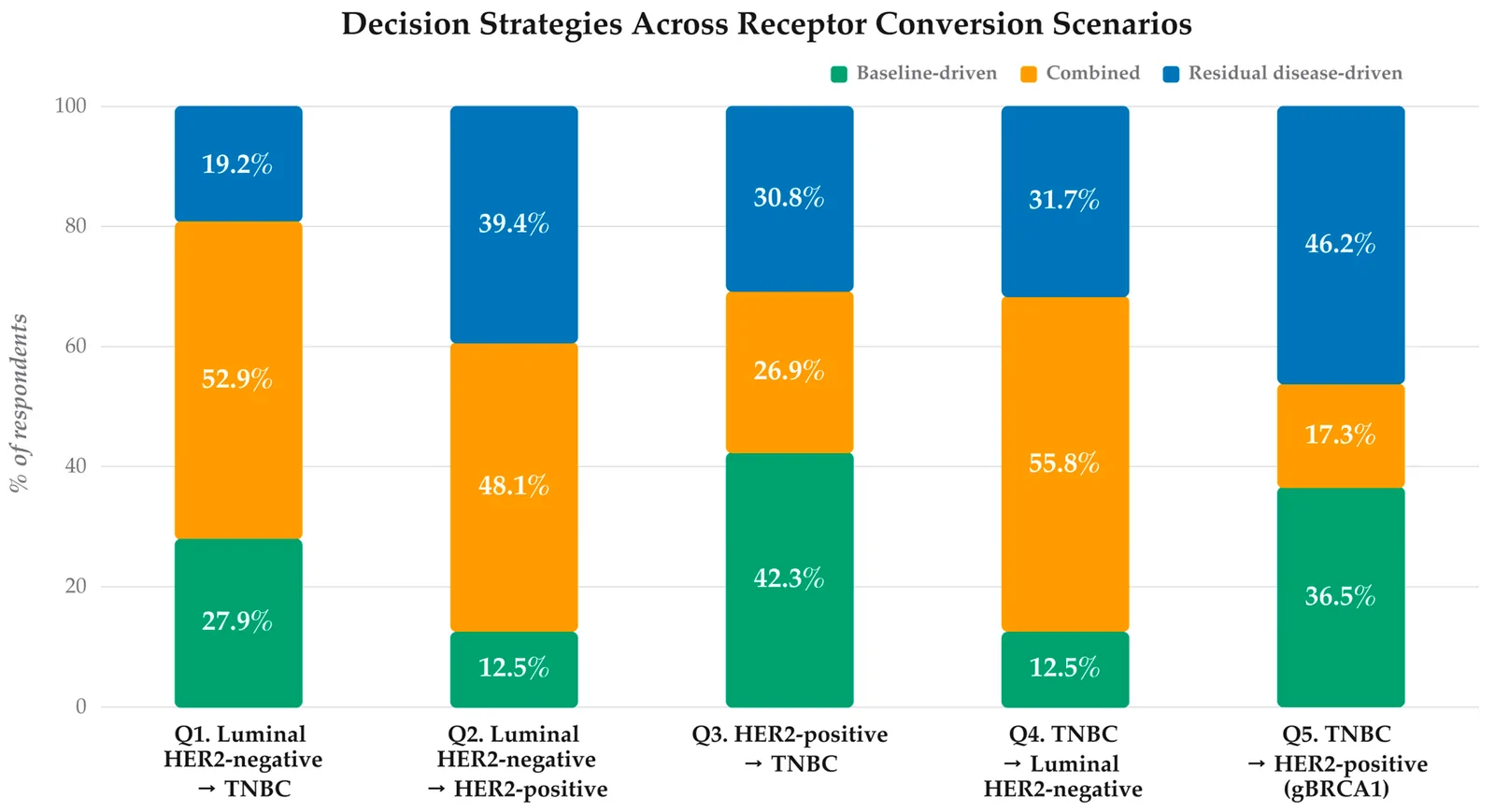
Clinical decision-making patterns across receptor conversion scenarios. Abbreviations: HER2, human epidermal growth factor receptor 2; TNBC, triple-negative breast cancer.

By comparison, residual disease-driven approaches were most frequently selected in TNBC to HER2-positive conversion in the context of a germline *BRCA* mutation (46.2%) and were least common in luminal HER2-negative to TNBC conversion (19.2%; Figure S4) (Figure 2, Table S1). Taken together, these findings indicate that treatment adaptation after receptor change was not uniform and varied according to the direction of receptor change and the perceived actionability of the residual disease phenotype.

## 4. Discussion

Post-neoadjuvant treatment adaptation following receptor conversion has become an increasingly relevant clinical challenge as biomarker reassessment is performed more frequently in routine practice. The principal finding of this study is not simply the presence of variability in clinical practice but the identification of three distinct patterns of post-neoadjuvant treatment decision-making following receptor conversion. Baseline-driven strategies based exclusively on initial tumor characteristics accounted for only 26.3% of responses overall. Despite limited guidance from current guidelines, most respondents reported adapting treatment decisions based on post-neoadjuvant receptor status in at least some scenarios. These findings suggest that receptor conversion is not only a biomarker issue but also a source of variable management in routine oncology practice.

Across clinical scenarios, treatment decisions were most frequently based on combined strategies integrating both baseline and residual disease characteristics, with residual disease-driven approaches also selected in a substantial proportion of cases. Overall, clinicians appeared to adopt pragmatic treatment strategies when evidence is limited, retaining elements of baseline-directed treatment while responding to residual disease biology. Emerging guidance from the College of American Pathologists (CAP), together with recommendations from the Spanish Society of Medical Oncology and the Spanish Society of Pathology (SEOM–SEAP) and recent Chinese Society of Clinical Oncology (CSCO) guidelines, supports consideration of biomarker reassessment in selected scenarios, reflecting an increasing recognition of tumor heterogeneity following neoadjuvant therapy [14,15,16]. In metastatic breast cancer, international guidelines recommend biopsy of an accessible metastatic lesion and reassessment of hormone-receptor and HER2 status, as discordance from the primary tumor may alter treatment selection [6,17,18]. In particular, receptor gain may expand therapeutic options. However, this approach cannot be directly extrapolated to residual early-stage disease, where post-neoadjuvant treatment remains partly anchored in the pretreatment phenotype and evidence from trials based on baseline biology. Meta-analyses have shown that receptor conversion is associated with clinically meaningful differences in patient outcomes, supporting the biological relevance of biomarker reassessment [19,20]. However, evidence remains insufficient to define how receptor changes should guide treatment selection. Our findings suggest that clinicians integrate baseline and residual disease biology pragmatically when prospective evidence is lacking.

Decision-making patterns varied according to the direction of receptor change. Clinicians appeared to view receptor gain and receptor loss as biologically and therapeutically distinct events: acquisition of actionable targets, such as HER2 positivity, favored residual disease-driven or combined strategies, whereas HER2 loss was more often associated with baseline-driven approaches.

These observations also highlight a gap between biomarker assessment and its clinical implementation. Although approximately half of respondents reported routine receptor reassessment in residual disease, this practice was more commonly reported by clinicians working in dedicated breast cancer units, high-volume centers, and those with more than 10 years of clinical experience. However, reassessment did not consistently translate into treatment adaptation. Notably, clinicians who did not routinely perform reassessment still selected residual disease-driven strategies in some hypothetical scenarios, suggesting a distinction between current practice infrastructure and clinical reasoning under survey conditions.

The use of additional pathological parameters, such as tumor grade and Ki-67, further indicates that treatment adaptation is influenced by a broader biological context rather than receptor status alone. Variability in the use of capecitabine in hormone receptor-positive residual disease similarly reflects inconsistent approaches to treatment intensification when evidence is limited.

Our findings extend prior reports demonstrating substantial variability in post-neoadjuvant biomarker assessment. Survey-based studies among pathologists have shown inconsistent practices regarding receptor reassessment, with one study including 23 pathologists reporting routine retesting in approximately 65% of cases, while another survey with 32 respondents found systematic biomarker reassessment in only 41% [21,22]. Importantly, these studies focused primarily on pathology workflows and reporting practices, without addressing how such findings influence clinical decision-making. By surveying oncologists actively involved in breast cancer care, the present study adds insight into how receptor changes are translated into reported treatment preferences when formal guidance is limited.

Existing literature on receptor change in residual disease provides little insight into its impact on treatment decision-making, as most studies have focused primarily on the frequency, patterns, and prognostic implications of biomarker changes rather than their relevance to real-world oncology care delivery [11,23,24]. To our knowledge, this is the first national survey specifically evaluating how medical oncologists incorporate receptor conversion into post-neoadjuvant treatment decision-making. Rather than documenting variability alone, our study provides a structured framework for describing how clinicians integrate baseline and residual disease biology when evidence is lacking.

In the current post-neoadjuvant setting, evidence directly guiding treatment adaptation following receptor conversion remains limited. The most clinically relevant evidence comes from the KATHERINE trial, which established adjuvant T-DM1 for patients with residual tumor after neoadjuvant therapy for baseline HER2-positive breast cancer [25]. Importantly, an additional analysis showed that T-DM1 benefit was maintained even when residual disease tested HER2-negative [26]. For most other receptor conversion scenarios, including receptor gain and loss outside this setting, no prospective evidence or guideline-based recommendations are available, and treatment decisions rely on extrapolation and individual clinical judgment. This uncertainty may help explain the frequent use of combined treatment strategies in our survey.

These findings have direct implications for clinical practice. In the absence of standardized guidance, patients with similar clinical characteristics may receive different adjuvant treatment recommendations depending on physician preference or institutional practice. This variability underscores the need for clearer guidance and prospective evidence to support post-neoadjuvant treatment adaptation. Beyond describing current practice, the proposed three-strategy framework identified in this study provides a pragmatic structure for future prospective studies evaluating biomarker-guided treatment strategies after neoadjuvant therapy.

This study has several limitations. First, it reflects self-reported preferences and responses to hypothetical clinical scenarios rather than actual treatment decisions, which may be influenced by institutional protocols, multidisciplinary tumor board input, and patient preferences. Second, participation was voluntary and may have introduced selection bias. Because the survey was distributed through multiple, partly overlapping recruitment channels, the number of eligible medical oncologists involved in breast cancer care who received the questionnaire could not be determined, precluding reliable calculation of the response rate. Importantly, treatment preferences differed substantially across the five clinical scenarios, suggesting scenario-specific clinical reasoning rather than uniform response patterns. Third, the questionnaire was expert-developed and internally reviewed but not formally validated. Although the scenarios and treatment options were informed by contemporary international guidelines and therapeutic principles, the predefined choices necessarily simplified complex decision-making and may have introduced framing bias, omitted relevant alternatives, or failed to reflect differences in treatment availability across healthcare systems. Accordingly, the findings should be considered an exploratory assessment among participating oncologists rather than a population-representative estimate of national clinical practice. Future larger surveys applying this framework in broader and more diverse samples of oncologists could further assess the reproducibility and generalizability of these findings. Nevertheless, respondents represented diverse experience levels and practice settings, supporting the relevance of the observed decision-making patterns.

## 5. Conclusions

This national survey identified three distinct patterns of post-neoadjuvant treatment decision-making following receptor conversion. Baseline-driven strategies accounted for only one quarter of all scenario-specific responses, whereas most respondents incorporated residual tumor biology into at least some treatment decisions despite limited guideline direction. These findings suggest that current clinical practice is driven largely by clinical judgment rather than evidence-based recommendations. The proposed three-strategy framework provides a practical foundation for future prospective studies evaluating biomarker-guided treatment adaptation after receptor conversion.

## Figures and Tables

**Table 1:** Framework for classification of baseline-driven, combined, and residual disease-driven treatment strategies across receptor conversion scenarios.

Scenario (Q)	Treatment Option Selected	Treatment Strategy Classification	Rationale for Classification
Q1 (Luminal HER2-negative → TNBC)	ET	Baseline-driven	Maintains treatment according to the baseline luminal phenotype
	ET + CDK4/6i	Baseline-driven	Escalates baseline luminal therapy without considering TNBC conversion
	Capecitabine + ET	Combined	Preserves endocrine therapy while adding treatment for residual TNBC
	Capecitabine + ET + CDK4/6i	Combined	Integrates baseline luminal and residual TNBC biology
	Capecitabine	Residual disease-driven	Treatment based solely on the residual TNBC phenotype
Q2 (Luminal HER2-negative → HER2-positive)	ET + CDK4/6i	Baseline-driven	Maintains treatment according to the baseline luminal phenotype
	ET + CDK4/6i + trastuzumab	Combined	Integrates baseline endocrine therapy with HER2-targeted treatment
	ET + CDK4/6i + dual anti-HER2	Combined	Combines baseline luminal treatment with intensified HER2-directed therapy
	ET + trastuzumab	Residual disease-driven	Treatment adapted according to acquired HER2 positivity
Q3 (HER2-positive → TNBC)	T-DM1	Baseline-driven	Maintains treatment according to baseline HER2-positive disease
	Capecitabine + T-DM1	Combined	Combines baseline HER2-directed therapy with TNBC-directed treatment
	Capecitabine	Residual disease-driven	Treatment based solely on the residual TNBC phenotype
Q4 (TNBC → Luminal HER2-negative)	Capecitabine ± pembrolizumab	Baseline-driven	Maintains treatment according to baseline TNBC biology
	Capecitabine ± pembrolizumab + ET	Combined	Integrates baseline TNBC treatment with endocrine therapy
	ET	Residual disease-driven	Treatment adapted according to acquired hormone receptor positivity
	ET + CDK4/6i	Residual disease-driven	Treatment based on the residual luminal phenotype
Q5 (TNBC → HER2-positive with germline *BRCA* mutation)	Olaparib	Baseline-driven	Maintains *BRCA*-directed treatment according to baseline TNBC
	Olaparib + pembrolizumab	Baseline-driven	Continues baseline TNBC-directed systemic therapy
	Olaparib + pembrolizumab + trastuzumab	Combined	Integrates baseline TNBC treatment with HER2-targeted therapy
	Olaparib + trastuzumab	Residual disease-driven	Treatment adapted according to acquired HER2 positivity while preserving *BRCA*-targeted therapy

Treatment options were classified according to the predominant biological information guiding the therapeutic decision: baseline tumor characteristics (baseline-driven), residual disease biomarkers (residual disease-driven), or integration of both sources of information (combined). The classification was developed using contemporary therapeutic principles and available international guideline recommendations and was intended solely as an analytical framework rather than an assessment of clinical appropriateness. Abbreviations: CDK4/6i, cyclin-dependent kinase 4 and 6 inhibitor; ET, endocrine therapy; HER2, human epidermal growth factor receptor 2; *mBRCA*—*BRCA* mutation; T-DM1, trastuzumab emtansine; TNBC, triple-negative breast cancer.

**Table 2:** Respondent and institutional characteristics.

Characteristic	No. (%)
Certified Breast Cancer Unit Affiliation	
Yes	65 (62.5)
No	39 (37.5)
Annual number of newly diagnosed breast cancer cases at the respondent’s institution	
<50 cases	21 (20.2)
50–250 cases	23 (22.1)
250–500 cases	21 (20.2)
500–1000 cases	27 (26.0)
>1000 cases	12 (11.5)
Years of clinical experience	
1–2 years	10 (9.6)
2–5 years	15 (14.4)
5–10 years	24 (23.1)
10–20 years	31 (29.8)
>20 years	24 (23.1)

**Table 3:** Receptor reassessment practices and reported influence on treatment decisions.

Characteristic	No. (%)
Receptor reassessment	
Routine receptor reassessment in residual disease	
Yes	55 (52.9)
No	49 (47.1)
Site of receptor reassessment	
Residual breast tumor only	17 (16.3)
Axillary lymph nodes only	9 (8.7)
Both breast and axillary lymph nodes	29 (27.9)
Not applicable	49 (47.1)
**Treatment adaptation**	
Use of residual disease assessment in treatment decision-making	
Yes	47 (45.2)
Sometimes	29 (27.9)
No	28 (26.9)
Use of tumor grade in residual disease in decision-making	
Yes	70 (67.3)
No	34 (32.7)
Use of Ki-67 in residual disease in decision-making	
Yes	71 (68.3)
No	33 (31.7)
Use of tumor grade and Ki-67 to guide capecitabine use in luminal HER2-negative residual disease	
Yes	62 (59.6)
No—always use capecitabine in luminal HER2-negative residual disease	15 (14.4)
No—never use capecitabine in luminal HER2-negative residual disease	27 (26.0)

## Data Availability

The datasets generated and/or analyzed during the current study are available from the corresponding author upon reasonable request.

## Supplementary Materials

The following supporting information can be downloaded at https://www.mdpi.com/article/10.3390/jcm15187141/s1, Table S1: Distribution of reported treatment strategies across clinical scenarios. File S1: Survey. Questionnaire used in the national survey. Figure S1: Within-scenario comparisons of reported treatment strategy selection across receptor conversion scenarios. Numbers on the bars indicate the number of respondents selecting each strategy. Braces indicate pairwise comparisons between two strategies using McNemar’s test, and horizontal lines indicate overall comparisons across all three strategies using Cochran’s Q test. α indicates the significance level. Figure S2: Comparison of baseline-driven strategy selection across receptor conversion scenarios. Numbers on the bars indicate the number of respondents selecting the baseline-driven strategy in each scenario. Braces indicate pairwise comparisons between scenarios using McNemar’s test, and the horizontal line indicates the overall comparison across scenarios using Cochran’s Q test. α indicates the significance level. Figure S3: Comparison of combined strategy selection across receptor conversion scenarios. Numbers on the bars indicate the number of respondents selecting the combined strategy in each scenario. Braces indicate pairwise comparisons between scenarios using McNemar’s test, and the horizontal line indicates the overall comparison across scenarios using Cochran’s Q test. α indicates the significance level. Figure S4: Comparison of residual disease-driven strategy selection across receptor conversion scenarios. Numbers on the bars indicate the number of respondents selecting the residual disease-driven strategy in each scenario. Braces indicate pairwise comparisons between scenarios using McNemar’s test, and the horizontal line indicates the overall comparison across scenarios using Cochran’s Q test. α indicates the significance level.
