# Supplementary material for: Clinical Decision-Making After Receptor Conversion Following Neoadjuvant Therapy in Breast Cancer: A Three-Strategy Framework
Source: J Clin Med. 2026 Sep 15;15(18):7141. doi: 10.3390/jcm15187141 (PMC13607326; doi:10.3390/jcm15187141)
Supplement: Supplementary file 1 [file jcm-15-07141-s001.zip › File S1-Survey_questionnaire_final.pdf]

**Survey Questionnaire**

## Assessment of Receptor Changes After Neoadjuvant Therapy in Breast Cancer Patients in Poland

Dear Colleagues,

We are observing a growing need to better understand current clinical practice regarding the assessment of receptor status in residual disease following neoadjuvant therapy for breast cancer. These results are increasingly influencing treatment decisions, while at the same time, there are no consistent national recommendations in this area.

The aim of this survey is to collect information on current practices and expert opinions. The results will be used to support the development of recommendations by the Polish Society of Clinical Oncology (PTOK).

The survey is anonymous.

Thank you for your time and for your valuable contribution to establishing shared clinical standards.

## Section 1. Respondent and Institutional Characteristics

**1. Do you work in a center with a certified Breast Cancer Unit (BCU)?**

☐ Yes
☐ No

**2. Approximately how many new breast cancer patients are treated annually in your center?**

☐ <50
☐ 50–250
☐ 250–500
☐ 500–1000
☐ >1000

**3. In which city do you currently practice?.........................**

**4. How many years have you been treating breast cancer patients?**

☐ 1–2 years
☐ 2–5 years
☐ 5–10 years
☐ 10–20 years
☐ >20 years

## Section 2. Institutional Practice

**5. Is receptor status (ER, PgR, HER2) routinely assessed in residual disease in your center?**

☐ Yes

☐ No

**6. If yes, from which material is receptor status assessed?**

☐ Residual primary tumor only

☐ Metastatic lymph nodes only

☐ Both residual primary tumor and involved lymph nodes

☐ Not applicable

**7. Does adjuvant treatment decision-making in your center depend on receptor status in residual disease?**

☐ Yes

☐ No

☐ Sometimes

## Section 3. Clinical Scenarios

**8. What treatment would you recommend for a patient with <u>TNBC</u> at baseline and <u>luminal (ER+/HER2−)</u> residual disease after neoadjuvant therapy?**

☐ Capecitabine ± pembrolizumab

☐ Capecitabine ± pembrolizumab + endocrine therapy

☐ Endocrine therapy alone

☐ Endocrine therapy + CDK4/6 inhibitor

**9. What treatment would you recommend for a patient with <u>non-luminal HER2-positive (ER−/HER2+)</u> breast cancer at baseline and residual <u>TNBC</u> after neoadjuvant therapy?**

☐ T-DM1

☐ Capecitabine + T-DM1

☐ Capecitabine

**10. What treatment would you recommend for a patient with <u>luminal HER2-negative (ER+/HER2−)</u> breast cancer at baseline and <u>HER2-positive (ER+/HER2+)</u> residual disease after neoadjuvant chemotherapy with ≥4 positive lymph nodes?**

☐ Endocrine therapy + CDK4/6 inhibitor

☐ Endocrine therapy + trastuzumab

☐ Endocrine therapy + CDK4/6 inhibitor + trastuzumab

☐ Endocrine therapy + CDK4/6 inhibitor + trastuzumab + pertuzumab

**11. What treatment would you recommend for a patient with TNBC at baseline and HER2-positive (ER−/HER2+) residual disease after chemoimmunotherapy in a BRCA1 mutation carrier?**

☐ Olaparib

☐ Olaparib + pembrolizumab

☐ Olaparib + trastuzumab

☐ Olaparib + pembrolizumab + trastuzumab

**12. What treatment would you recommend for a patient with cT3N1 luminal HER2-negative breast cancer at baseline and residual TNBC after neoadjuvant chemotherapy, without a BRCA1/2 mutation?**

☐ Endocrine therapy

☐ Endocrine therapy + CDK4/6 inhibitor

☐ Capecitabine

☐ Capecitabine + endocrine therapy

☐ Capecitabine + endocrine therapy + CDK4/6 inhibitor

## Section 4. Additional Decision Factors

**13. When deciding on postoperative treatment, do you take into account tumor grade (G) in residual disease and any differences between baseline and postoperative histopathological findings?**

☐ Yes

☐ No

**14. When deciding on postoperative treatment, do you take into account Ki-67 expression in residual disease and any differences between baseline and postoperative histopathological findings?**

☐ Yes

☐ No

**15. When deciding on the use of capecitabine in the adjuvant setting for patients with luminal breast cancer (ER+/HER2−) after neoadjuvant chemotherapy, do you consider tumor grade (G) or the Ki-67 index in residual disease?**

☐ Yes

☐ No – I routinely use capecitabine in residual disease in luminal breast cancer (ER+/HER2−)

☐ No – I do not use capecitabine in residual disease in ER+/HER2− breast cancer

**Thank you for your time and contribution!**
