# Supplementary material for: Clinical Decision-Making After Receptor Conversion Following Neoadjuvant Therapy in Breast Cancer: A Three-Strategy Framework
Source: J Clin Med. 2026 Sep 15;15(18):7141. doi: 10.3390/jcm15187141 (PMC13607326; doi:10.3390/jcm15187141)
Supplement: Supplementary file 1 [file jcm-15-07141-s001.zip › Supplementary Figures.pdf]

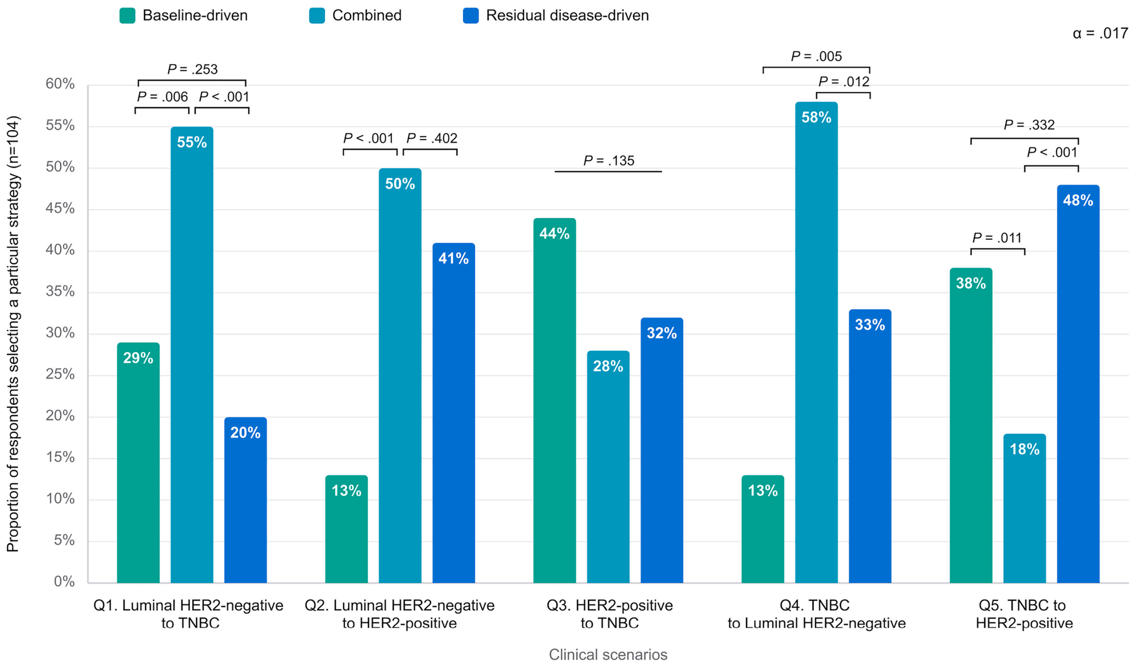

Figure S1. Within-scenario comparisons of reported treatment strategy selection across receptor conversion scenarios. Numbers on the bars indicate the number of respondents selecting each strategy. Braces indicate pairwise comparisons between two strategies using McNemar's test, and horizontal lines indicate overall comparisons across all three strategies using Cochran's Q test. α indicates the significance level.

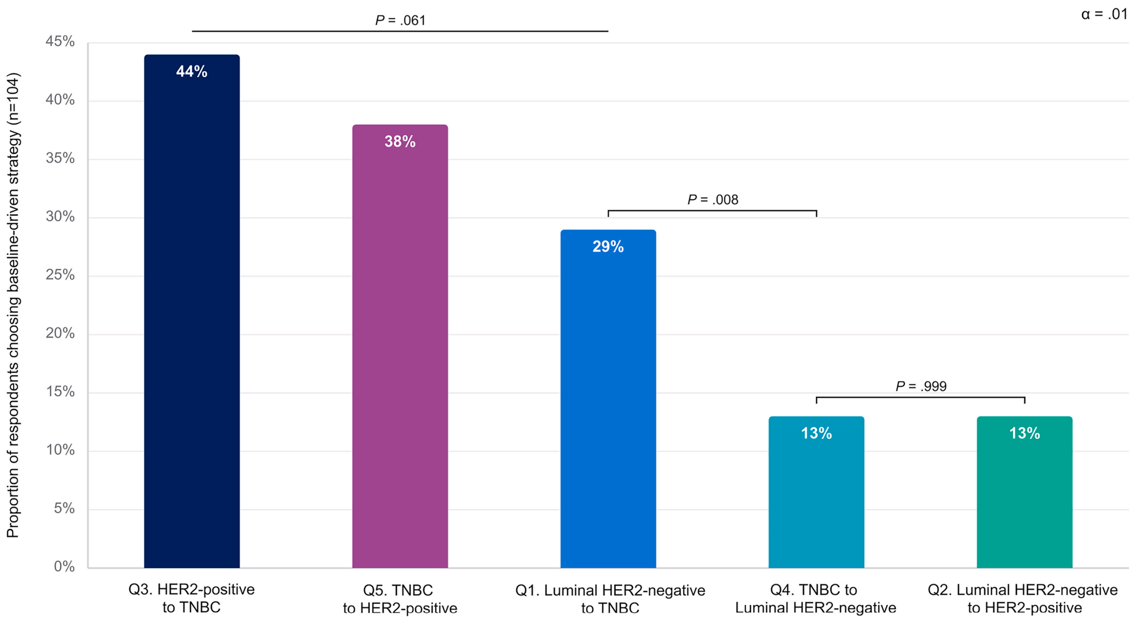

Figure S2. Comparison of baseline-driven strategy selection across receptor conversion scenarios. Numbers on the bars indicate the number of respondents selecting the baseline-driven strategy in each scenario. Braces indicate pairwise comparisons between scenarios using McNemar's test, and the horizontal line indicates the overall comparison across scenarios using Cochran's Q test. α indicates the significance level.

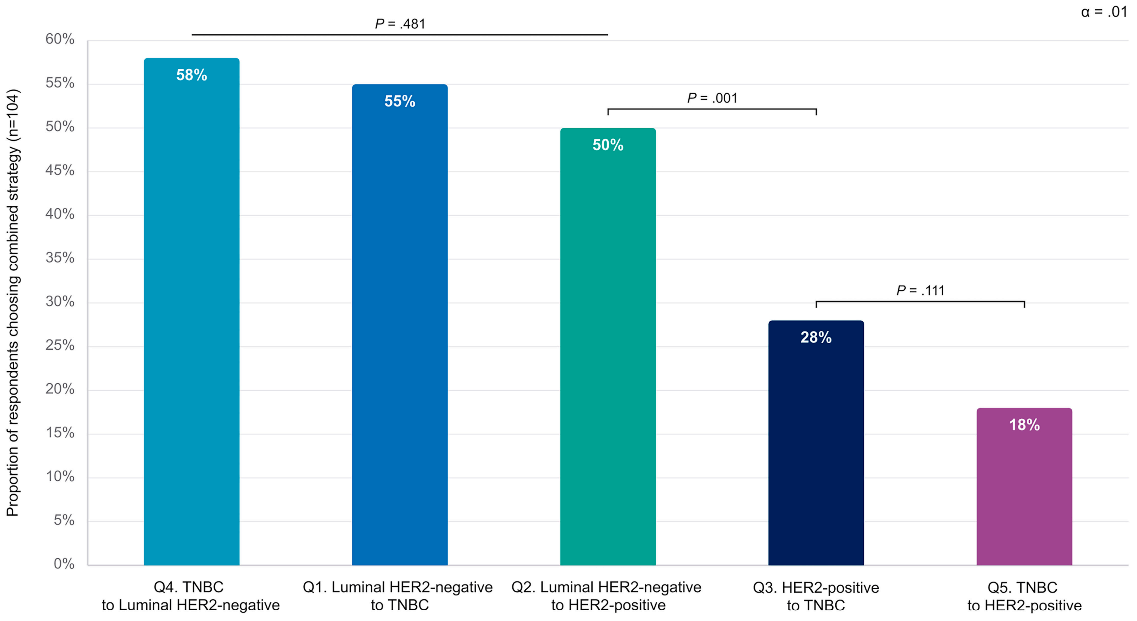

Figure S3. Comparison of combined strategy selection across receptor conversion scenarios. Numbers on the bars indicate the number of respondents selecting the combined strategy in each scenario. Braces indicate pairwise comparisons between scenarios using McNemar's test, and the horizontal line indicates the overall comparison across scenarios using Cochran's Q test. α indicates the significance level.

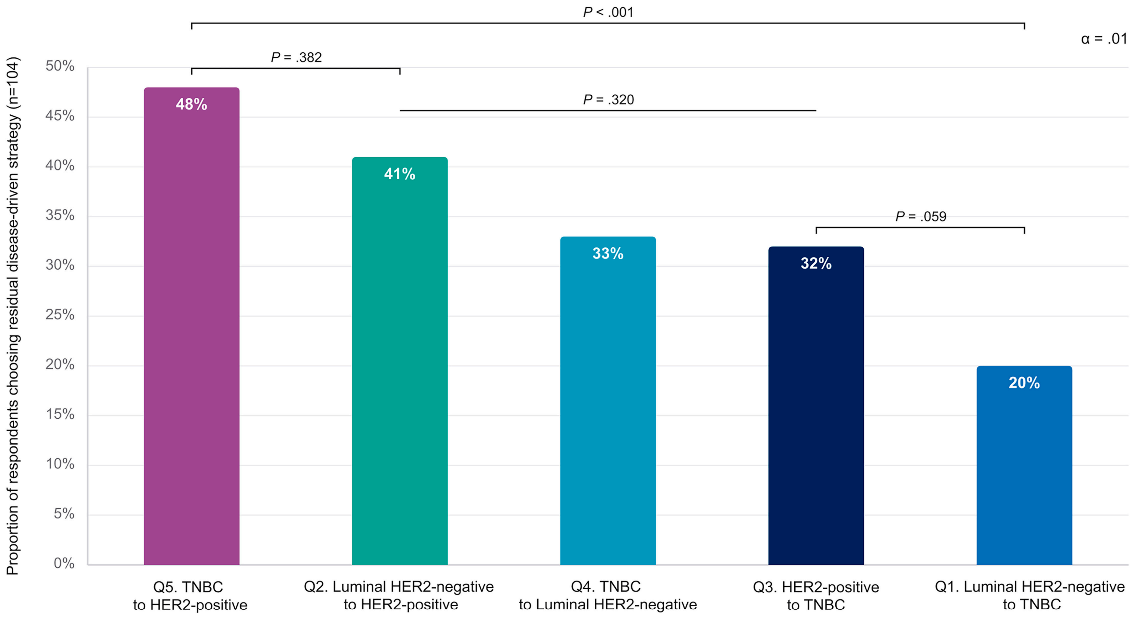

Figure S4. Comparison of residual disease-driven strategy selection across receptor conversion scenarios. Numbers on the bars indicate the number of respondents selecting the residual disease-driven strategy in each scenario. Braces indicate pairwise comparisons between scenarios using McNemar's test, and the horizontal line indicates the overall comparison across scenarios using Cochran's Q test. α indicates the significance level.
