# Supplementary material for: Clinical Decision-Making After Receptor Conversion Following Neoadjuvant Therapy in Breast Cancer: A Three-Strategy Framework
Source: J Clin Med. 2026 Sep 15;15(18):7141. doi: 10.3390/jcm15187141 (PMC13607326; doi:10.3390/jcm15187141)
Supplement: Supplementary file 1 [file jcm-15-07141-s001.zip › Supplementary Table_JCM_ReceptorConversion.pdf]

Table S1. Distribution of reported treatment strategies across clinical scenarios. Distribution of predefined reported treatment strategies (baseline-driven, combined, and residual disease-driven) across five case-based clinical scenarios reflecting different patterns of receptor conversion. Values are presented as numbers and percentages of respondents selecting each strategy within a given scenario.

Scenario	Baseline-driven, No. (%)	Combined, No. (%)	Residual disease-driven, No. (%)
Q1. Luminal HER2-negative → TNBC	29 (27.9)	55 (52.9)	20 (19.2)
Q2. Luminal HER2-negative → HER2-positive	13 (12.5)	50 (48.1)	41 (39.4)
Q3. HER2-positive → TNBC	44 (42.3)	28 (26.9)	32 (30.8)
Q4. TNBC → Luminal HER2-negative	13 (12.5)	58 (55.8)	33 (31.7)
Q5. TNBC → HER2-positive with germline BRCA mutation	38 (36.5)	18 (17.3)	48 (46.2)
**Overall strategy distribution**	**137 (26.3)**	**209 (40.2)**	**174 (33.5)**

Overall percentages are calculated across all scenario responses.

Abbreviations:

HER2, human epidermal growth factor receptor 2; TNBC, triple-negative breast cancer.
